# Quercetin Synergistically Enhances the Anticancer Efficacy of Docetaxel through Induction of Apoptosis and Modulation of PI3K/AKT, MAPK/ERK, and JAK/STAT3 Signaling Pathways in MDA-MB-231 Breast Cancer Cell Line

**DOI:** 10.22088/IJMCM.BUMS.10.1.11

**Published:** 2021-05-22

**Authors:** Amir Safi, Esfandiar Heidarian, Reza Ahmadi

**Affiliations:** *Clinical Biochemistry Research Center, Basic Health Sciences Institute, Shahrekord University of Medical Sciences, Shahrekord, Iran.*

**Keywords:** Apoptosis, breast neoplasms, cell survival, combined modality therapy, docetaxel, quercetin

## Abstract

Docetaxel is widely used in the treatment of metastatic breast cancer. However, its effectiveness is limited due to chemoresistance and its undesirable side effects. The combination of chemotherapeutic agents and natural compounds is an effective strategy to overcome drug resistance and the ensuing inevitable toxicities. Quercetin is a natural flavonoid with strong antioxidant and anticancer activities. This study aimed to evaluate the cytotoxic and modulatory effects of combined docetaxel and quercetin on the MDA-MB-231 human breast cancer cell line. The cell viability was assessed by MTT assay. The induction of apoptosis was examined using flow cytometry. The role of *p53* in the apoptotic process was evaluated *via* qRT-PCR. The levels of BAX, BCL2, ERK1/2, AKT, and STAT3 proteins were measured by Western blot analysis. The results showed that the single-agent treatment with docetaxel or quercetin leads to a decrease in the viability of the MDA-MB-231 cells at 48 h. Furthermore, the combination of docetaxel (7 nM) and quercetin (95 μM) displayed the greatest synergistic effects with a combination index value of 0.76 accompanied by the up regulation of *p53* and a significant increase in BAX level, as well as decrease in the levels of BCL2, pERK1/2, AKT, and STAT3 proteins (P < 0.05). The concomitant use of docetaxel and quercetin leads to the cell growth inhibition associated with the induction of apoptosis and inhibition of cell survival. Therefore, this study provides a promising therapeutic approach to enhance the efficacy of docetaxel in a less-toxic manner.

Triple-negative breast cancer (TNBC) represents the most malignant subtype of breast cancer (BC), and is characterized by a lack of expression of three receptors commonly found on BC cells (1). Chemotherapy remains the mainstay for the treatment of TNBC due to the lack of targeted therapies (2). Docetaxel is a chemotherapeutic drug that belongs to taxanes, a class of anti-neoplastic agents commonly prescribed for metastatic BC (3). It acts through binding to the beta-tubulin subunit of the microtubules, leading to the stabilization of tubulin polymerization, resulting in cell cycle arrest at the G2/M phase and inhibition of mitosis (4). Although docetaxel is an effective drug, toxicities and some adverse effects may limit its administrable dose (5). Also, since cancer may have hundreds of gene mutations and many pathways cross talking with one another in tumor development, most probably it will not be possible to control cancer by targeting a single or few signaling pathways. As a result, chemoresistance may develop during treatment, which is a major reason for the failure of chemotherapy drugs (6). Tumor cells can acquire resistance to apoptosis by downregulation or mutation of proapoptotic proteins such as BCL2 associated X (BAX) or by the expression of anti-apoptotic proteins like B-cell lymphoma 2 (BCL2). The expression of both *BAX* and *BCL2* is regulated by *p53* tumor suppressor gene (7). Furthermore, activating protein kinase B (PKB, also known as AKT), extracellular signalregulated protein kinase (ERK1/2), and signal transducer and activator of transcription (STAT3) as proliferation and survival signaling mediators cause tumor cell chemor-esistance (8-10). Recently, a promising approach for cancer treatment has been drug combination therapies. Combination therapy may be able to prevent toxic reaction on normal cells while simultaneously producing a synergistic or additive effect by targeting different pathways. It has been clear that this strategy can be developed as an effective therapy to impede the growth of BC cells (11). Natural products have been shown to selectively target cancer cells, with minimum toxicity in normal cells (12). Quercetin, a natural polyphenolic flavonoid compound, has been reported to exhibit a broad range of encouraging pharmacological properties (13). The anticancer effects of quercetin have been extensively studied in many *in vitro* and *in vivo* cancer models (14-16). Quercetin induces apoptosis and cell death in BC cells through multiple mechanisms (17). In this study, we aimed at investigating the possible synergistic cytotoxic and modulatory effects of docetaxel in combination with quercetin on human BC cell line, MDA-MB-231.

## Materials and methods

**Cell culture**

The MDA-MB-231 cells were purchased from the Pasteur Institute (Tehran, Iran), and cultured in RPMI-1640 medium (Gibco, USA) enriched with 10% FBS (Gibco, USA) and 1% pen/strep (Gibco, USA) at 37°C under a humidified atmosphere containing 5% CO_2_ (18).

**Cell viability/proliferation assay**

Cell viability was determined using a colorimetric 3-(4,5-Dimethylthiazol-2-yl)-2, 5-diphenyltetrazolium bromide (MTT) assay. MDA-MB-231 cells were seeded in a 96-well plate (5000 cells/well) and incubated overnight. Subsequently, cells were treated with different concentrations of docetaxel (80 mg/4 mL, Sanofi-Aventis, France) (0-50 nM) and quercetin (Q4951-10G, Sigma-Aldrich, USA) (0-200 μM in 0.1% dimethyl sulfoxide (DMSO) (Sigma-Aldrich, USA) solution) for 48 h. After treatment, the medium was removed and the cells were incubated with 10 μL MTT (Sigma-Aldrich, USA) solution (5 mg/ml) for 4 h at 37ºC in a dark place. Then 150 μL DMSO was added to each well to dissolve the formazan crystals. A microplate reader (Stat Fax-2100, USA) was used to read the absorbance of each well at 570 nm with the background subtraction of 630 nm. The survival rate was calculated according to the following formula: percentage of cell viability = [(absorbance of treatment)/ (absorbance of control)] × 100. These experiments were conducted in triplicate. The results were used to determine the concentrations of docetaxel and quercetin required to inhibit the growth of MDA-MB-231 cells by 50, 40, 30, 20, and 10% (i.e., the IC_50_, IC_40_, IC_30_, IC_20_, and IC_10_ values).

**Determination of docetaxel and quercetin syne-rgism**

Based on the IC values, MTT assay was conducted using four different combinations of docetaxel and quercetin (i.e., IC_40_ docetaxel + IC_10_ quercetin, IC_30_ docetaxel + IC_20_ quercetin, IC_20_ docetaxel + IC_30_ quercetin, and IC_10_ docetaxel + IC_40_ quercetin). The cells were seeded in a 96-well plate (5000 cells/well) with an incubation period of 24 h. The medium was subsequently removed and replaced with fresh medium containing the test compound, followed by an incubation period of 48 h. The cells were then incubated with MTT solution (5 mg/mL) for 4 h, and the resulting formazan precipitate was dissolved in 150 μL DMSO. The absorbance of each well was then measured at 570 and 630 nm using the microplate reader. At least three independent experiments were carried out (19). Synergism was evaluated using CompuSyn software (Combo SynInc, City, State, USA), and the combination index (CI) and drug reduction index (DRI) values were determined. CI<1 indicates synergism, CI= 1 indicates additive effect, and CI> 1 indicates antagonism.The combination with the smallest CI was selected to perform other tests. The DRI also specifies how many folds of dose-reduction are allowed for each drug in synergistic combinations (20).

**Apoptosis assay**

Cell deaths (apoptosis and necrosis) were examined by FITC-conjugated annexin V and propidium iodide (PI) staining (BD Bioscience, USA). The cells were seeded into a 6-well plate (2×10^5^ cells/well) and incubated overnight. Then, they were treated with docetaxel (7 nM), quercetin (95 μM), and the combination of docetaxel with quercetin (7 nM and 95 μM, respectively) for 48 h. The cells were collected by trypsinization and washed with PBS. Afterwards, they were resuspended in binding buffer, and subsequently stained with annexin V-FITC and PI according to the manufacturer’s protocol. After an incubation of 25 min at room temperature in a dark place, flow cytometric analysis was done using a Partec ML flow cytometer (Germanu), and the results were analyzed by PartecFloMax and Flow Jo software (Treestar, Ashland, OR, USA) (21).

**Quantitative reverse transcription PCR (qRT-PCR)**

Cells were seeded in the 6-well plate (3×10^5^ cells/well), and after a night they were treated with docetaxel (7 nM), quercetin (95 μM), and the combination of docetaxel with quercetin (7 nM and 95 μM, respectively) for 48 h. Total RNA of all culture groups was extracted drawing on TRIzol reagent (Invitrogen Life Technologies, USA) based on the manufacturer’s protocol. The quantity and quality of RNA were measured using a NanoDrop spectrophotometer 2000 (Thermo Scientific, USA). Then, mRNA (1 µg) was transcribed into cDNA in line with the manufacturer's guidelines of cDNA synthesis kit (Yekta Tajhiz Azma, Iran). QRT-PCR was performed using SYBR Green PCR Master Mix (Yekta Tajhiz Azma, Iran) with a Rotor-Gene 3000 real-time thermal cycler (Corbett Research, Australia) in the presence of specific primers for *p53* (forward: 5'CCCATCCTCACCATCATCACA C-3', reverse: 5'GCACAAACACGCACCTCAA AG3', and β-actin (forward: 5'TCATGAAGTGT GACGTGGACATC3', reverse: 5'CAGGAGGAG CAATGATCTTGATCT3'). Relative mRNA expre- ssion levels were calculated by the 2^−∆∆CT^ method (22).

**Western blot**

The cells were cultured in 6 cm dishes (6×10^5^ cells/dish) and after a night, they were treated with docetaxel (7 nM), quercetin (95 μM), and the combination of docetaxel with quercetin (7 nM and 95 μM, respectively). Following an incubation period of 48 h, the cells were washed with PBS and lysed using 8 M urea solution.The supernatant containing proteins were collected, and protein concentration was quantified by the Bradford method (23). The protein samples were mixed with an equal volume of loading buffer and were boiled for 5 min at 98°C. Denatured proteins were separated by 10% sodium dodecyl sulfate-polyacrylamide gel electrophoresis (SDS-PAGE) and were electro-transferred onto polyvinylidene difluoride (PVDF) membrane. Then, membranes were placed in a blocking solution with 5% BSA for 1 h. The membranes were washed 3 times in TBS-Tween buffer (containing 10 mM Tris with pH 7.4, 100 mM NaCl, and 0.1 mM Tween 20) for 10 min, and they were incubated with primary BAX (Elabscience Biotechnology Co., China), BCL2 (fElabscience Biotechnology Co., China), AKT, ERK1/2, STAT3, and β-actin (as an internal control) antibodies (Abcam, UK) according to the manufacturer’s protocols at 4°C overnight. Then, the membranes were washed with TBS-Tween buffer 3 times for 10 min and were incubated with secondary antibody at room temperature for 2 h. After washing the membranes 3 times for 10 min in TBS-Tween buffer, bands were visualized using Li-Cor C-DiGit Blot Scanner (Lincoln, USA) (24).

**Data analysis**

Statistical analysis was performed using SPSS software version 26.0 (IBM SPSS, Chicago, IL, USA). All data are expressed as mean ± standard deviation (SD) of three independent experiments. Differences between the experimental and control groups were assessed using the Kruskal-Wallis test followed by Dunn's multiple comparisons test. P values less than or equal to 0.05 were considered to indicate statistically significant differences.

## Results

**The inhibitory effects of docetaxel and quercetin alone and in combination on cell growth**

**Fig. 1:**
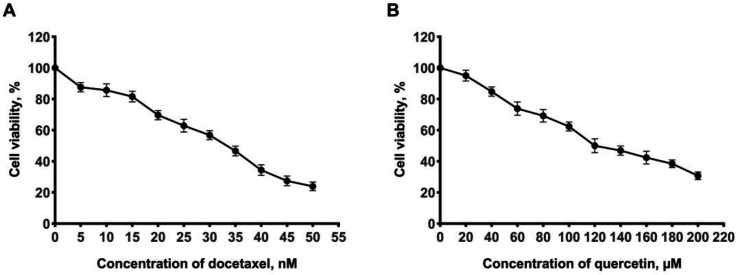
**Anti-proliferative effects of docetaxel and quercetin.** A) Docetaxel; B) Quercetin. The results are expressed as the mean ± SD of three independent experiments

The anti-proliferative effects of docetaxel and quercetin were determined by MTT assay. The treatment of the cells with docetaxel or quercetin alone resulted in a dose-dependent decrease in the viability of the MDA-MB-231 cells at 48 h, in which higher concentrations resulted in lower levels of cell viability (Figure 1). The IC_50_ values for docetaxel and quercetin were 33 nM and 125 µM, respectively. The results of the four different combinations of docetaxel and quercetin are also shown in Table 1. Combination 4 (docetaxel 7 nM + quercetin 95 μM) had the lowest CI value equal to 0.76 and induced the greatest reduction in cell viability indicating that it exhibits the strongest inhibitory impact amongst the four tested combinations.

**Fig. 2:**
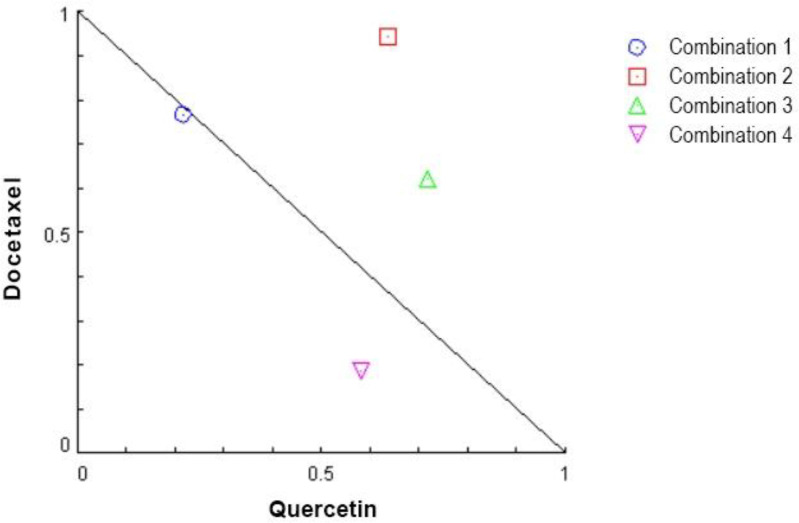
**Isobologram plot for the combined use of docetaxel and quercetin in treatment of MDA-MB-231 cells.** Diagonal experimental combination data points, represented by dots located lower left, on the diagonal line, or upper right, indicate synergism, additively, and antagonism, respectively

The results of the isobologram were consistent with those of the CI values, and were indicative of the presence of the best synergistic effect for combination 4 at 48 h (Figure 2). The DRI values also supported the presence of synergistic effects for combination 4, which had the highest DRI value for docetaxel (Table 2). The DRI values showed that the IC_50_ dose of docetaxel could be reduced 7-fold when it was used concomitantly with quercetin in combination 4. These findings demonstrated that combination 4 showed the best synergistic effects among all other combinations tested in this study at 48 h. This combination was, therefore, selected for the subsequent experiments.

**Table 1:** Viability and combination index (CI) values of MDA-MB-231 after 48 h treatment with docetaxel and quercetin combinations

**Combination number**		**Combination dosage**				**Cell viability (%)**		**CI value**	
			**Docetaxel (nM)**		**Quercetin (µM)**	
**1**		25 nM (IC_40_)		30 µM (IC_10_)		51.39		0.98	
**2**		19 nM (IC_30_)		50 µM (IC_20_)		68.43		1.58	
**3**		15 nM (IC_20_)		70 µM (IC_30_)		62.27		1.34	
**4**		7 nM (IC_10_)		95 µM (IC_40_)		46.07		0.76	

The results are expressed as mean ± SD of three separate experiments.

**Table 2:** Dose reduction index (DRI) for combining docetaxel and quercetin

**Combination number**		**Combination dosage**				**DRI (Docetaxel)**		**DRI **	
			**Docetaxel (nM)**		**Quercetin (µM)**		**(Quercetin)**	
**1**		25 nM (IC_40_)		30 µM (IC_10_)		1.30363		4.60660	
**2**		19 nM (IC_30_)		50 µM (IC_20_)		1.06078		1.56570	
**3**		15 nM (IC_20_)		70 µM (IC_30_)		1.61259		1.38767	
**4**		7 nM (IC_10_)		95 µM (IC_40_)		5.36975		1.72206	

**Induction of apoptosis increased by the com-bined docetaxel and quercetin treatment**

**Fig. 3:**
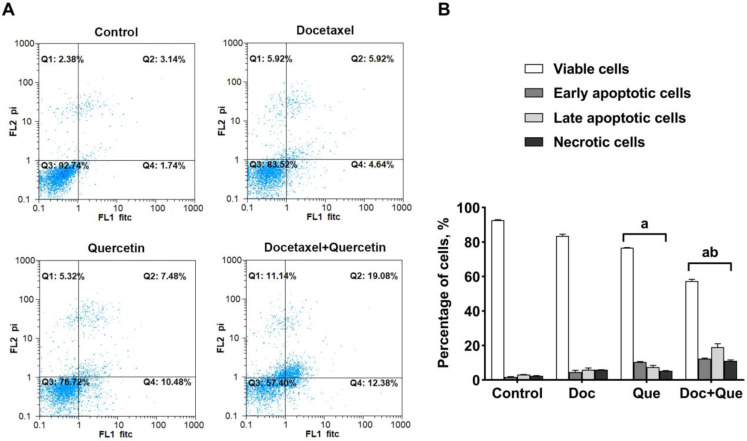
**Effects of docetaxel, quercetin, and their combination on the apoptosis of MDA-MB-231 cells.** A) Dot-plots from flow cytometry illustrate apoptotic and necrotic status; B) The percentage of alive, apoptotic, and post apoptotic/necrotic cells. The results are expressed as the mean ± SD of three separate experiments. a: P < 0.05 *vs*. control cells; b: P < 0.05 *vs.* docetaxel treated cells. Doc: docetaxel; Que: quercetin

The flow cytometry was performed on the treated and untreated control cells to ascertain whether the growth inhibitory effects of combined treatment could be attributed to apoptosis. The results of flow cytometry show the percentage of apoptosis and necrosis of docetaxel and quercetin (Figure 3). The rates of apoptosis in MDA-MB-231 cells with a single treatment of docetaxel, quercetin, and their combined treatment were 10.56%, 17.96%, and 31.46%, respectively. Docetaxel in 

**Fig. 4.**
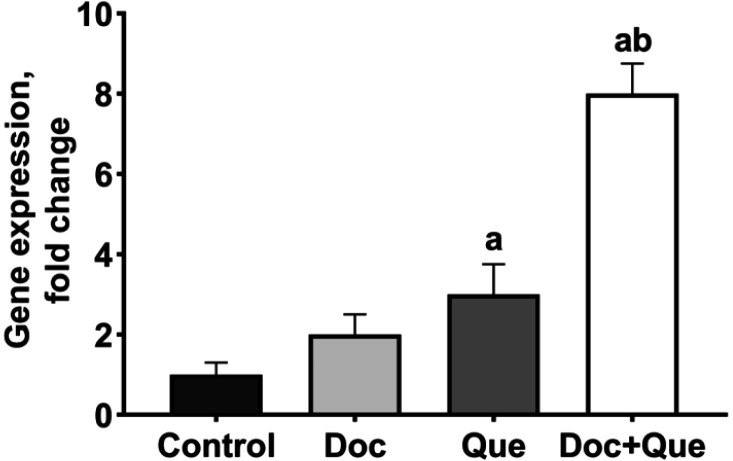
***p53*** **expression in the presence of docetaxel and quercetin alone, or their combination in MDA-MB-231 cells.** The expression of *p53* was normalized with β-actin as an internal standard. The results are expressed as the mean ± SD of three separate experiments. a: P<0.05 *vs*. control cells; b: P < 0.05 *vs.* docetaxel treated cells. Doc: docetaxel; Que: quercetin

combination with quercetin resulted in a significant increase (P <0.05) in apoptosis in comparison with untreated control cells.

**Combined treatment with docetaxel and querc-etin induces** ***p53*** **upregulation**

The findings of qRT-PCR are shown in Figure 4. The *p53* gene expression significantly increased about 8-fold and 3-fold in cells with combined treatment and single treatment with quercetin, respectively, in comparison with the untreated control cells (P<0.05). No significant differences were observed between single treatment with docetaxel and untreated control cells (P>0.05).

**Fig. 5:**
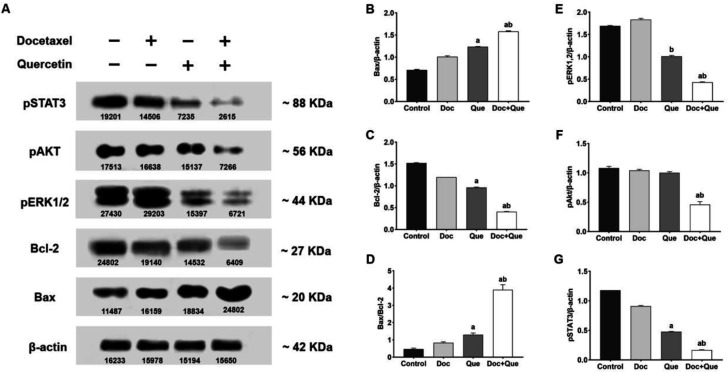
**Western blot analysis of** **BAX** **, BCL2, ERK1/2, AKT, and** **STAT** **3 proteins in** **MDA** **-MB-231 cells treated with docetaxel, quercetin, and their combination.** A) Western blots bands; B) Relative levels of BAX; C) Relative levels of BCL2; D) Ratio of BAX to BCL2; E) Relative levels of ERK1/2; F) Relative levels of AKT; G) Relative levels of STAT3. β-actin was used as an internal reference. a: P < 0.05 *vs*. control cells; b: P < 0.05 *vs.* docetaxel treated cells. Doc: docetaxel; Que: quercetin

**The combination of docetaxel and quercetin induces apoptosis and modulates cell survival pathways**

Western blot was used to measure the level of cell survival and apoptosis-related proteins in MDA-MB-231 cells after 48 h (Figure 5). The results of this analysis revealed that the cells exposed to the combined treatment had much lower levels of BCL2, AKT, ERK1/2, and STAT3 proteins (P<0.05). However, the level of BAX increased significantly following the combined treatment with docetaxel and quercetin in comparison with the untreated control cells, as well as the single-agent treatments (P <0.05).

## Discussion

The present study demonstrated that the combination of quercetin and docetaxel significantly enhances the therapeutic effects of docetaxel on MDA-MB-231 BC cells.

Docetaxel is a widely-used chemotherapeutic agent that targets metastatic BC, but prolonged use of this drug results in drug resistance and toxicity in cancer patients (5, 25). Enhanced efficacy of docetaxel along with its minimal side effects will provide remarkable benefits to improve survival and life quality of patients. Using combination therapy with natural compounds is a popular approach to safeguard against the potential consequences of using a chemotherapeutic agent alone. Previous studies have shown that quercetin has anti-proliferative and pro-apoptotic effects on BC and many other malignancies (16, 26). We have put forward the proposal herein to evaluate the synergistic effects of quercetin in combination with docetaxel on the proliferation of MDA-MB-231 cells. Our results demonstrate that quercetin restores sensitivity towards docetaxel in TNBC, and significantly suppresses tumor cell growth *in vitro *(Table 1).

The data obtained by annexin V-FITC/PI assay indicate that the synergistic effects of co-treatment of docetaxel and quercetin are caused by an increased apoptotic response (Figure 3) which is in agreement with a previously published research (27). This impact could be attributed to different modes of action used by docetaxel and quercetin.

The combined drug treatment with docetaxel and quercetin results in a significant increase in the expression of the *p53* gene, thus promoting apoptosis in MDA-MB-231 cells (Figure 4). *p53* tumor suppressor gene is often inactivated in cancer cells, and its down-regulation leads to cancer progression (28). Previous studies suggest that quercetin prompts *p53* upregulation, which was also observed in our findings (29). It has been proposed that the presence of functional *p53* facilitates the cytotoxic effects of docetaxel (30). It has also been reported that in non-small-cell lung cancer and prostate cancer cells, *p53* expression induces apoptosis and enhances chemotherapeutic cytotoxic effects of docetaxel which is in agreement with our findings (31, 32).

Upon activation of *p53*, the pro-apoptotic protein BAX is up-regulated (Figure 5B), while the anti-apoptotic protein BCL2 is down-regulated (Figure 5C) in MDA-MB-231 cells. In other words, our findings support the idea that the regulation of apoptosis-related proteins expression and increasing of the ratio of BAX/BCL2 (Figure 5D) are possible underlying mechanisms of combined treatment action of docetaxel and quercetin (33-35). Docetaxel triggers the phosphorylation, and the subsequent inactivation of BCL2 results in apoptosis (36, 37), while quercetin exerts its apoptosis stimulatory impact *via p53* activation which, in turn, leads to the upregulation of BAX and downregulation of BCL2 in tumor cells. Lu et al. found that combining docetaxel and quercetin could inhibit prostate cancer cells by changing the expression of BAX and BCL2 proteins, which is consistent with our findings (38).

Our findings also show that some mediators of PI3K/AKT, MAPK/ERK, and JAK/STAT3 signaling pathways are activated in the MDA-MB-231 cells (Figure 5A). These mediators act as transcription factors, and are involved in the dysregulation of death machinery and the progression of BC (39). This suggests that the acquisition of docetaxel resistance may be related to the activation of PI3K/AKT, MAPK/ERK, and JAK/STAT3 signaling pathways. 

Our findings reveal that docetaxel or quercetin alone does not exert remarkable effects on MDA-MB-231 cells, but their combination reduces the production of AKT (Figure 5F). The activation of AKT promotes the expression of survival transcription factors, and inhibits the pro-apoptotic factors like BCL2-associated agonist of cell death (BAD) and forkhead box protein O (FOXO) (40). The activated AKT phosphorylates BAD at Ser-136 residue and prompts it to dissociate itself from the BCL2/BCL-xL complex, ultimately inhibiting cell apoptosis and decreasing the chemo-sensitivity (41). Our findings are consistent with several previous studies in this regard. Li et al. and Shu et al. demonstrated that, in combination with doxorubicin treatment, quercetin down-regulates the expression of AKT (42, 43). Another study by Pozsgai et al. also indicated that quercetin in combination with temozolomide or irradiation could induce apoptosis by suppressing the PI3K/AKT signaling pathway by lowering the level of AKT in glioblastoma cells (44). The study by Li et al*. *also showed that the expression of Akt is reduced after using a combination of docetaxel and quercetin in 4T1 murine BC cells (45).

Indeed, our findings are indicative of an intense activation of ERK1/2 in the MDA-MB-231 cells (Figure 5E). Neither docetaxel nor quercetin has minimal effects on inhibiting the ERK/MAPK pathway; even the level of ERK1/2 is increased upon treatment with docetaxel. The activation of the ERK1/2 by taxanes is well described in previous studies (46, 47). Nevertheless, the combined use of docetaxel and quercetin synergistically suppresses ERK1/2 (Figure 5E). It has been posited that ERK1/2 promotes chemoresistance and cell survival by activating BCL2 and degrading pro-death proteins such as BCL2-interacting mediator of cell death (BIM), BCL2-modifying factor (BMF), and p53 upregulated modulator of apoptosis  (PUMA) (48, 49). In line with the above results, the study of Chen et al. reported that down regulation of ERK1/2 protein by a combination of quercetin and adriamycin inhibits cell proliferation, and promotes apoptosis in multidrug resistant leukemia K562 cells (50).

We also investigated the involvement of another transcription factor, STAT3, which is known to be activated in several types of tumors, especially those of BC (51). This regulatory protein provides a survival mechanism by regulating the expression of target genes involved in inflammation, cell proliferation, invasion, angiogenesis, and metastasis, thereby representing a major causative factor for drug resistance (52). Previous reports have demonstrated that cancer cells harboring aberrant STAT3 activities elevate levels of anti-apoptotic proteins such as BCL2, BCL-xL, and myeloid cell leukemia 1 (MCL1). Hence, cancer cells over-expressing activated STAT3 are more likely to become resistant to apoptosis (53, 54). Interestingly, our findings indicate that quercetin alone or its concomitant use with docetaxel reduces STAT3 protein level in MDA-MB-231 cells (Figure 5G). Moreover, our results are consistent with those of a study that has demonstrated that a combination of docetaxel with a STAT3 inhibitor leads to the induction of apoptosis *via* a remarkable decrease in expression levels of *BCL2*, *MCL1*, and surviving while increasing *BAX* mRNA levels (55).

In conclusion, the current findings have indicated that quercetin increases the sensitivity of MDA-MB-231 cells to docetaxel *via* inducing apoptosis and reducing cell survival. The enhanced expression of the *p53* gene, pro-apoptotic protein BAX, and concurrent reduced expression of anti-apoptotic protein BCL2, phosphorylated AKT, ERK1/2, and STAT3 have been recognized to be involved in the synergistic action of the docetaxel and quercetin. Therefore, these findings support the possibility of considering quercetin as a potential adjuvant drug or chemosensitizer for the treatment of TNBC. In this study, we did not assess other mechanisms that probably contribute to the efficacy of combined quercetin-docetaxel treatment on MDA-MB-231 BC cell line such as NF-κB, caspases activation, BCL-xL, p38 MAPK, p27, p21, and also autophagic pathways. Thus, we suggest that future studies focus on other possible mechanisms of combined quercetin-docetaxel treatment.
